# Nicotine Enemas for Active Crohn's Colitis: An Open Pilot Study

**DOI:** 10.1155/2008/237185

**Published:** 2008-03-27

**Authors:** J. R. Ingram, J. Rhodes, B. K. Evans, G. A. O. Thomas

**Affiliations:** ^1^Department of Gastroenterology, Cardiff and Vale NHS Trust, University Hospital of Wales, Heath Park, Cardiff CF14 4XW, UK; ^2^St Mary's Pharmaceutical Unit, Quadrant Centre, Cardiff Business Park, Llanishen, Cardiff CF14 5RA, UK

## Abstract

*Background*. Smoking has a detrimental effect in Crohn's disease (CD), but this may be due to factors in smoking other than nicotine. Given that transdermal nicotine benefits ulcerative colitis (UC), and there is a considerable overlap in the treatment of UC and CD, the possible beneficial effect of nicotine has been examined in patients with Crohn's colitis. *Aims*. To assess the efficacy and safety of nicotine enemas in active Crohn's colitis. *Patients*. Thirteen patients with active rectosigmoid CD; 3 patients were excluded because they received antibiotics. *Methods*. Subjects were given 6 mg nicotine enemas, each day for 4 weeks, in an open pilot study. At the beginning and end of the trial, a Crohn's disease activity index (CDAI) score was calculated, sigmoidoscopy was performed, and haematological inflammatory markers measured. *Results*. Mean CDAI decreased from 202 to 153—the score was reduced in 6 patients, unchanged in 3, and increased in one. Frequency of bowel movements decreased in 8 patients and the sigmoidoscopy grade was reduced in 7. Mean C-reactive protein decreased from 22.0 to 12.3 mg/L. There were no withdrawals due to adverse events. *Conclusions*. In this relatively small study of patients with active Crohn's colitis, 6 mg nicotine enemas appeared to be of clinical benefit in most patients. They were well tolerated and safe.

## 1. INTRODUCTION

It is a remarkable epidemiological observation that whilst ulcerative colitis (UC) is related to nonsmoking [1–4], the opposite applies to Crohn's disease (CD).** Patients with CD are more often smokers compared with the general population [5], and smoking has an adverse effect on the course of their disease [6]. Several mechanisms for this could be relevant; components of tobacco smoke, such as oxidizing chemicals, which, unlike nicotine, have prothrombotic effects, could exacerbate microvasculature abnormalities and ischaemia of the bowel wall [7]. It has also been suggested that CD could be caused by an impaired host response to luminal bacteria; this, in turn, could be exacerbated by the immunosuppressive effects of smoking on macrophages [8, 9]. It is likely that different mechanisms are responsible for the “opposite effects” of smoking in CD and UC, which are in many other respects similar diseases. The effects of *smoking* should not be considered synonymous with *nicotine*. Nicotine, as opposed to smoking, may have a beneficial effect in patients with Crohn's colitis, given that there is a considerable overlap in the treatments for the two conditions and nicotine has been shown to be of benefit in UC. Although the specific mechanisms underlying this effect remain unclear, nicotine has a number of actions that could be potentially beneficial, including effects on the immune system [10] and gut motility [11].

A recent Cochrane Review [12] has confirmed benefit from transdermal nicotine in active UC: a meta-analysis of two eligible randomized placebo-controlled trials [13, 14] so far performed showed that after 4 to 6 weeks treatment, 19 of 71 patients treated with transdermal nicotine were in remission compared to 9 of 70 given placebo (odds ratio 2.56, 95% confidence interval 1.02–6.45). A nicotine enema has also been developed and found to be of benefit when given as additional therapy in two uncontrolled pilot studies in active distal UC [15, 16], but not in a recent randomised controlled trial [17]. A phase I-II trial of delayed release oral nicotine has shown promise [18] but a controlled trial is awaited.

The aim of this open pilot study was to examine the efficacy and safety of nicotine enemas in active distal Crohn's colitis.

## 2. MATERIALS AND METHODS

### 2.1. Patients

Patients with CD, based on the clinical, endoscopic, and histological features of Lennard-Jones' criteria [19], were recruited from the gastroenterology outpatient department of a single centre; the key selection criteria were clinical and sigmoidoscopic evidence of active disease in the rectosigmoid region. Although the presence of CD in other regions of the gastrointestinal tract was permitted, the patient's principal clinical problem had to relate to active distal colitis.** Patients were not enrolled if they were current smokers, had other unstable medical problems, were pregnant or lactating, had used enemas in the previous week, had changed their CD therapy with mesalazine, steroids, or antibiotics within the last 2 weeks, or had changed immunosuppressive therapy with azathioprine in the previous 3 months.** The dosage of all concomitant medications was kept unchanged during the study period.

### 2.2. Study design

This was an open pilot study of 4 weeks' duration in which nicotine enemas were given as additional therapy—subjects continued their conventional treatment without change during the study period. Each 100 mL liquid enema contained 6 mg of nicotine complexed with 400 mg of the high molecular weight polyacrylic acid carbomer, Carbopol 974P (Goodrich, UK), as already described [20]. At the time of enrolment, a record was made of the patient's symptoms of colitis including stool frequency, abdominal pain, general well being, any complications of CD and urgency of defaecation; the latter was graded as none, mild, moderate, or severe enough to cause incontinence. Although Crohn's colitis may be patchy, sigmoidoscopy with a biopsy of the most inflamed area was also performed by the same investigator in each case.**Patients were also asked to complete an inflammatory bowel disease quality of life questionnaire (SIBDQ) [21] and blood tests were taken for a full blood count, liver and renal function, and inflammatory markers.

During the study, patients kept a diary of their bowel symptoms and any adverse events (AEs). The patients were assessed at the end of the 4-week trial period, or at premature withdrawal, or at any other time at the patient's request. The initial assessments were repeated by the same physician. Sigmoidoscopy with a biopsy, again from the most inflamed area, was repeated.

### 2.3. Outcome measures

The main outcome measures used were clinical improvement as measured by Crohn's disease activity index (CDAI) [22], and changes in bowel frequency and urgency of defaecation. Urgency was included because it is a useful guide to the severity of inflammation in distal colitis. At sigmoidoscopy, the severity of inflammation of the worst affected area was graded visually according to the system described by Dick et al. [23]. The biopsy specimens were stained with haematoxylin and eosin and graded by one histopathologist (GTW) according to the Truelove and Richards system [24] adapted for Crohn's colitis; see Table 1.

### 2.4. Ethical considerations

This study was approved by the Bro Taf Local Research Ethics Committee.

## 3. RESULTS

Of the 13 patients recruited, 3 were excluded because they were also given antibiotics for a chest infection, a middle ear infection, and recurrence of a finger infection during the study period. Demographic details of the remaining 10 patients are in Table 2; 7 were male and the mean age was 52 years. Seven patients were exsmokers whilst the remainder had never smoked.**The mean duration of disease relapse was 45 weeks and in 4 patients was greater than 99 weeks. Half of the patients were on concomitant oral 5-ASAs, 2 were on prednisolone (10 mg) and 2 were on azathioprine; several patients had been intolerant of thiopurines. Only one patient had recently taken enemas, asacol foam. The mean baseline CDAI score was 202 (SD 80, range 73 to 348).** In addition to their colonic involvement, one patient also had small bowel CD. Three patients had disease limited to the left side of the colon.

### 3.1. Efficacy

**Figure 1:**
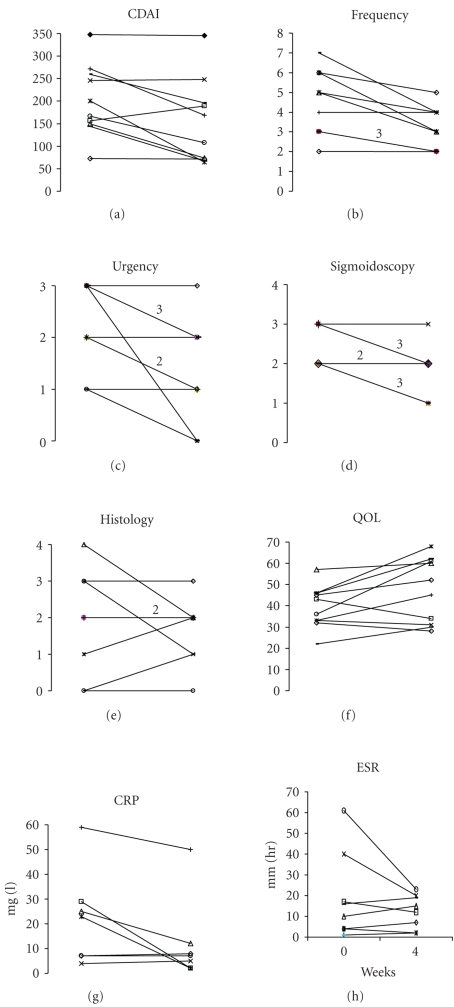
*Outcome measures.* Response of 10 patients with active Crohn's colitis given 6 mg nicotine enemas daily for 4 weeks. Where 2 or 3 patients had the same baseline and end values, this is indicated above the connecting line, which is drawn more boldly. CDAI is Crohn's disease activity index. Frequency is the number of stools/24 hours. Urgency of defaecation is graded: 0 = none, 1 = mild, 2 = moderate, and 3 = severe enough to cause incontinence. Sigmoidoscopic score is graded 0, normal to 4, fulminant disease. Histological scores are based on acute inflammatory activity: grade 0, no polymorphs to grade 4, florid acute inflammation with polymorphs and ulceration.** Histology data is given for only 8 patients because the end-of-study biopsies of 2, both with baseline scores of 2, were insufficient for analysis.**QOL is a score of quality of life as measured by the short inflammatory bowel disease questionnaire [21].**Paired data were available for CRP in 7 patients and for ESR in 8 patients.

Patient 10 withdrew from the study after only 2 weeks due to failure to respond to treatment, but was kept in the intention-to-treat analysis; she had the highest CDAI score (348) at baseline; all other patients completed 4 weeks' treatment. Changes in the main outcome measures for each patient are in Figure 1.** The mean CDAI score decreased from 202 to 153—the score was reduced in 6 patients, unchanged in 3, and increased in one patient. The frequency of bowel movements each day was reduced in 8 patients and unchanged in 2, and the urgency to defaecate was reduced in 7 patients, in one case from severe to nil, and was unchanged in the other 3. The sigmoidoscopy score improved in 7 patients and was unchanged in 3, but changes in histology showed no clear trend with a wide variation in the baseline scores.**One of the two patients taking oral steroids improved sufficiently to consider a gradual reduction of the dose after the study.

The mean SIBDQ score increased from 39 to 47.**Mean levels of the inflammatory markers C-reactive protein (CRP), erythrocyte sedimentation rate (ESR), and fibrinogen all fell from 22.0 mg/L, 19.1 mm/h, and 4.4 g/L, respectively, to 12.3 mg/L, 12.5 mm/h, and 4.0 g/L, respectively, whilst the mean white cell count was unchanged at 8.4 × 10^9^/L and the mean platelet count increased slightly from 310 to 320 × 10^9^/L.** Renal function and liver function tests were unaffected.

The effect of disease location on outcome was briefly considered. The only patient with ileal disease improved during the study, with a CDAI reduction from 150 to 74. Of the 3 patients with disease restricted to the left colon, one improved, one remained the same, and the other deteriorated; the respective changes in CDAI score were from 144 to 67, from 348 to 345, and from 156 to 189. **In terms of the duration of disease relapse, of the 4 patients who had been in relapse for more than 99 weeks, 3 improved, with CDAI changes from 272 to 169, from 200 to 64, and from 150 to 74, whilst one remained the same, with a CDAI change from 73 to 71.

### 3.2. Safety

**Figure 2:**
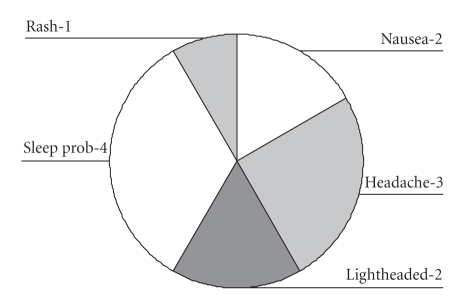
*
Adverse events during study.* Adverse events experienced during nicotine enema treatment with the number of patients affected in each case.**“Sleep prob” refers to disturbed sleep or vivid dreams.**The “rash” was a 2 cm diameter erythematous maculopapular lesion on the forearm of a patient which resolved spontaneously.

Eight of the 10 patients experienced an adverse event but none were classified as serious.**In total, 12 AEs were reported (Figure 2) and 11 of these were thought to be related to nicotine, the other being a localised skin rash that resolved spontaneously.**Two-thirds of the AEs occurred on commencement of treatment and were temporary, lasting 2 days or less.**The most common AE was difficulty sleeping, which included vivid dreams, reported by 4 patients. The other AEs were lightheadedness, nausea, and headache.

## 4. DISCUSSION

These are the first observations of topical nicotine in patients with active Crohn's colitis. In the 10 patients, there was a CDAI reduction in 6 and an increase in one; the mean score decreased from 202 to 153. Frequency of bowel movements was reduced in 8 patients, urgency to defaecate was reduced in 7, and the sigmoidoscopy score improved in 7; the mean SIBDQ score increased from 39 to 47. Clinical improvement was noted in patients who also had disease in locations other than the rectosigmoid, including the one patient with small bowel disease.**Three of the 4 patients with chronically active disease for more than 99 weeks improved. There were no serious AEs and no premature withdrawals due to intolerance.

A relatively small open pilot study of this nature can only provide preliminary data on efficacy and safety. Hence, although our observations suggested improvement in most of the patients, and certainly no overall deterioration, the findings will need to be corroborated by larger randomised controlled studies. Formal tests of significance were not performed because, in this relatively small study, they may have been misleading.** Initially, it was planned to recruit 20 patients to the study, but recruitment was slow for two main reasons; 10 patients with active Crohn's colitis, who were identified as suitable for entry to the study, were current smokers and therefore excluded. In addition, because the study preparation was an enema, those patients whose principal symptoms were related to left-sided active colitis were targeted. However, identifying such a homogenous group proved hard, as a number of patients screened had to be excluded because they had symptoms that related primarily to other sites of disease involvement. This may have reduced potential sources of error from our study, but it will probably make it difficult to recruit sufficient numbers to achieve a larger study of homogeneous patients, as defined by the Vienna classification [25]. For this small pilot study, full Vienna classification of patients was not performed and subgroup analyses were avoided.

It is noteworthy that methods used to assess the response of Crohn's colitis to treatment have some limitations. The patchy nature of Crohn's colitis means sigmoidoscopy and histology findings should be interpreted with caution. The separate recording and analysis of stool frequency and urgency of defaecation was an attempt to assess some of the more troublesome symptoms of distal colitis, although a contribution from more proximal disease cannot be excluded. This was used in addition to the global assessment provided by the CDAI, the more conventional composite scoring system of disease activity that contains systemic features less relevant to this study. Two of the 10 patients had a CDAI score less than 150 at study entry, which might imply disease remission; however, all patients had rectosigmoid inflammation on sigmoidoscopy and had symptoms of active distal disease.

The negative association between smoking and CD raises questions about whether topical nicotine is an appropriate treatment for patients with Crohn's colitis. However, this was justified on the basis that the treatments for UC and CD are very similar, and transdermal nicotine has been found to provide benefit in UC. It is possible that the detrimental effect of smoking on CD is mediated through mechanisms independent of nicotine; these could, for example, include exacerbation of underlying ischaemia of the bowel wall at the microvascular level [7] due to prothrombotic oxidizing chemicals present in tobacco smoke. It has also been suggested that CD could be caused by an impaired host response to luminal bacteria; this, in turn, could be exacerbated by the immunosuppressive effects of smoking on macrophages [8, 9]. These effects could mask any benefit from nicotine in Crohn's colitis. It is also possible that the response to nicotine differs between ileal and colonic diseases. Rectal enema treatment of the transmural inflammation of Crohn's colitis is not well described in the literature, however it is used in clinical practice.

Possible mechanisms for a therapeutic effect of topical nicotine in distal colitis include alterations in colonic motility.** Nicotine has been shown to reduce tone and muscular activity in the sigmoid colon [11], an effect mediated primarily through nitric oxide that is released by nicotine [26]. Another potential mechanism is via a reduction in mucosal tumour necrosis factor (TNF*α*), a key cytokine in the generation of inflammation in CD [27]. Nicotine has been found to reduce the secretion of TNF*α* from macrophages, by activation of *α*7 nicotinic acetylcholine receptors [28].

Our pilot study has shown that, in the 10 patients with active Crohn's colitis, 6 mg nicotine enemas were associated with clinical improvement in the majority of patients, they were safe and well tolerated, and did not appear to worsen the condition. Larger randomised controlled trials are needed to confirm these observations and longer-term outcome data would also be of interest.

## Figures and Tables

**Table 1:** *Histological grade of inflammation.* The histological grade of inflammation present in biopsies taken from the most inflamed area of the rectosigmoid region at sigmoidoscopy, adapted by GTW for Crohn's colitis from the system described by Truelove and Richards [24] for ulcerative colitis.

Grade of inflammation	Description
0	No polymorphs
1	Small number of polymorphs in the lamina propria with minimal cryptitis
2	Focal cryptitis with crypt rupture or affecting groups of two or more adjacent crypts
3	Florid diffuse polymorph infiltrate with crypt abscesses
4	Florid acute inflammation with ulceration

**Table 2:** *Baseline characteristics of 10 patients with Crohn's colitis.* Extent of disease: 1 = proctitis, 2 = sigmoid colon, 3 = descending colon, 4 = transverse colon, 5 = ascending colon, 6 = pancolitis, 7 = ileal, 8 = perianal. “Recent enemas” refers to any steroid or 5 aminosalicylate (5-ASA) enemas taken from 1 to 3 weeks before entry into the study. All concomitant medication doses given are the daily amounts, the only oral steroid taken was prednisolone, aza = azathioprine. CDAI = Crohn's disease activity index.

No.	Age (years)	Sex	Exsmoker	Disease extent	Duration relapse (weeks)	Recent enemas	5-ASA (g)	Oral steroids (mg)	Aza (mg)	Baseline CDAI score
1	41	M	−	1,2,3,4	>99	−	−	−	−	73
2	27	M	+	1	1	−	−	−	−	156
3	52	F	+	6,7	>99	−	−	10	−	150
4	59	M	+	2,5	7	−	3.2	−	150	246
5	73	M	+	6	>99	−	2.4	10	−	200
6	38	M	−	6,8	9	−	−	−	−	167
7	59	F	+	2,3,4	>99	−	1.2	−	−	272
8	79	M	+	3,4	30	−	−	−	−	259
9	27	M	−	1	1	−	3.0	−	−	144
10	62	F	+	2,3	5	foam	2.4	−	50	348
